# Adaptive NKG2C^+^ NK cells in cytomegalovirus seropositive individuals predominantly lack NKR‐P1A receptor expression

**DOI:** 10.1002/eji.202451562

**Published:** 2025-05-27

**Authors:** Mohamad Basem Alkassab, Fareeha Ajmal Shaikh, Caroline Hamm, Mir Munir A. Rahim

**Affiliations:** ^1^ Department of Biomedical Sciences University of Windsor Windsor Ontario Canada; ^2^ Windsor Regional Cancer Centre Windsor Regional Hospital Windsor Ontario Canada; ^3^ Division of Medical Oncology Western University London Ontario Canada

**Keywords:** CD161, cytomegalovirus, inhibitory receptor, NK cell, NKR‐P1A

## Abstract

The impact of cytomegalovirus (CMV) infection in shaping natural killer (NK) cell receptor (NKR) repertoire highlights the importance of NKRs in immunity against CMV. NKR‐P1A (CD161) is an inhibitory NKR, whose expression is lost during CMV infection, but its role in NK cell responses during CMV infection is not known. Here, we show selective expansion of adaptive NKG2C^+^ NK cells lacking NKR‐P1A receptor (NKR‐P1A^‒^) due to their increased activation and proliferation compared with NKR‐P1A^+^ NK cells in CMV‐infected individuals. *In vitro* stimulation of PBMCs showed similar inherent proliferative capacity in both NKR‐P1A^+^ versus NKR‐P1A^‒^ NK cells in steady state and upregulation, but not loss of NKR‐P1A receptor expression, in sorted NK cells. Furthermore, CMV infection induced differential gene expression profiles in NKR‐P1A^+^ versus NKR‐P1A^‒^ NK cells, and only NKR‐P1A^‒^ NK cells exhibited transcriptome signatures associated with adaptive NK cells in CMV‐infected individuals. This study further highlights the impact of CMV infection in shaping NK cell receptor repertoire and exclusion of NK cells that express the NKR‐P1A receptor from the adaptive NKG2C^+^ NK cell population that expands during CMV infection.

## Graphical abstract

Expansion of the adaptive NKG2C^+^ NK cells during cytomegalovirus (CMV) infection is restricted to the subset lacking NKR‐P1A receptor. This is associated with increased proliferation of NKR‐P1A^‒^, but not NKR‐P1A^+^, NK cells in CMV‐infected individuals.

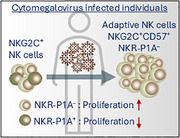

## 1 INTRODUCTION

Natural killer (NK) cells are lymphocytes that represent the innate arm of the immune system but also exhibit adaptive features such as clonal expansion and long‐term immunological memory [1, 2]. NK cells possess cytotoxic functions, in which they release granules containing perforin and granzymes to induce apoptosis in target cells [1] and produce immunomodulatory cytokines, including interferon‐γ (IFN‐γ), which can aid in the activation of T cell responses [3]. NK cells are one of the early responders to viral infections. Patients with NK cell deficiency are highly prone to recurrent infections by herpesviruses [4]. Similarly, NK cell depletion in mice led to increased susceptibility to viral infections and higher mortality, demonstrating the key role of NK cells in immunity against viral infections [5, 6].

NK cell functions are regulated via the integration of signals from activating and inhibitory cell surface receptors [7]. NK cell receptors aid in the detection and elimination of virus‐infected cells. Viral glycoproteins or stress‐induced ligands can be detected by the activating receptors, such as NKG2D [8, 9] and natural cytotoxicity receptors (NKp44 and NKp46), leading to NK cell activation and killing of infected cells [10, 11]. In parallel, viral proteins are known to interfere with antigen presentation by down‐modulating class I major histocompatibility (MHC‐I) proteins to escape detection by the CD8^+^ T‐cells [12, 13]. However, killer cell immunoglobulin (Ig)‐like receptors (KIRs) and leukocyte Ig‐like receptors (LIRs) on NK cells can detect reduced MHC‐I expression in infected cells [14, 15], leading to “missing‐self” recognition and target cell elimination [16]. Antibody‐dependent cell cytotoxicity (ADCC) is another mechanism used by NK cells to recognize and kill virally infected cells with the help of their CD16 Fc receptor [17].

NK cell receptors play important roles in cytomegalovirus (CMV) pathogenesis in both humans and mice. Several studies have shown the imprinting of human CMV infection on NK cell receptor repertoire and expansion of NK cells expressing NKG2C receptor, implicating it in virus recognition and immune responses against CMV [18, 19]. A leader peptide from CMV UL40 protein was found to be presented in complex with human leukocyte antigen‐E (HLA‐E:pUL40) on CMV‐infected cells that could engage the inhibitory NKG2A receptor for NK cell inhibition [20, 21]. The activating NKG2C receptor recognizes the same HLA‐E:pUL40 complex to circumvent NK cell inhibition [22]. This results in the expansion of NK cells expressing the activating NKG2C receptor in CMV‐infected individuals [18, 19]. These interactions are further affected by a polymorphism in UL40 peptide, modulating the affinity of interactions with NKG2 receptors, and thereby, NK cell response [23]. In mice, the m157 protein encoded by the murine CMV (MCMV) genome engages the inhibitory Ly49C and Ly49I receptors for NK cell inhibition [24]. In MCMV‐resistant mouse strains, such as C57BL/6 (B6), the activating Ly49H receptor has evolved to recognize m157 protein, leading to NK cell activation and killing of MCMV‐infected cells [24, 25, 26]. Similarly, MCMV m12 protein can interact with the inhibitory NKR‐P1B, and activate NKR‐P1C and NKR‐P1A receptors, demonstrating a role for NKR‐P1 receptors in modulating NK cell responses during MCMV infection in mice [27, 28].

NKR‐P1 receptors are type‐II transmembrane C‐type lectin‐like proteins that recognize other C‐type‐lectin‐like proteins encoded by genetically linked genes in the natural killer gene complex (NKC) [29]. NKR‐P1 receptor genes are conserved across many species [30, 31]. The NKR‐P1 receptor family in humans is composed of the inhibitory NKR‐P1A (CD161), which recognizes lectin‐like transcript‐1 (LLT‐1) protein, and activating NKp80 (KLRF1), NKp65 (KLRF2) receptors, which bind to activation‐induced C‐type lectin (AICL) and keratinocyte‐associated C‐type lectin (KACL) proteins, respectively [29, 32, 33]. NKR‐P1A is considered one of the earliest markers of NK cell development in humans [34]. In addition to its expression in a subset of mature NK cells, NKR‐P1A is found on γδ T cells, NK‐T cells, mucosal‐associated invariant T (MAIT) cells, and on approximately 25% of peripheral T cells with an effector/memory phenotype [7]. Loss of NKR‐P1A expression in NK cells, NK‐T cells, and T cells has been reported in CMV seropositive individuals [34, 35]. Single‐cell multi‐omic analysis of NK cells has also shown the *KLRB1* gene, which encodes the NKR‐P1A receptor, to be one of the frequently downregulated genes in NK cells from CMV seropositive individuals [37, 38]. However, the mechanisms of NKR‐P1A receptor downregulation and the role of this receptor in anti‐CMV immunity are not known.

In the current study, we analyzed peripheral blood mononuclear cells (PBMC) from healthy CMV seropositive and hematopoietic stem cell transplant (HSCT) patients harboring active CMV infection to show greater activation, proliferation, and expansion of NKR‐P1A^‒^ NK cells relative to NKR‐P1A^+^ NK cells in CMV‐infected individuals. This phenotype was restricted to the CMV‐specific NKG2C^+^ and the adaptive CD57^+^NKG2C^+^ NK cell populations. Analysis of publicly available single‐cell RNA sequence datasets showed that only NKR‐P1A^‒^, but not NKR‐P1A^+^, NK cells in CMV seropositive individuals possess transcriptome signatures associated with adaptive NK cells that expand during CMV infection. These findings demonstrate that expansion of NKG2C^+^ NK cells during cytomegalovirus infection is restricted to the subset lacking NKR‐P1A receptor further highlighting the impact of CMV infection in shaping NK cell receptor repertoire.

## 2 Materials and Methods

### 2.1 Study Participants

Two cohorts of blood samples were included in the study: (i) peripheral blood from healthy donors (83 participants) received from the Canadian Blood Services, and (ii) peripheral blood from HSCT patient donors (7 participants) collected at the Windsor Regional Hospital (WRH). Healthy donors included 61.5% CMV seronegative and 38.5% CMV seropositive (CMV serology test described below) participants. Amongst the CMV seronegative participants, 27% were females of 21–79 years and 73% were males of 20–80 years. Amongst the CMV seropositive participants, 28% were females of 29–79 years and 72% were males of 20–80 years. CMV‐negative HSCT patient donors included three male (23, 41, and 41 years) and one female (17 years) participants. Blood samples were collected approximately 3–8 months after receiving HSCT. CMV‐positive HSCT patient donors included one male (64 years) with a viral load of 8.59 × 10^3^ IU/mL (CMV DNA copies/mL blood), and two female (61 and 64 years) participants with viral loads of 5.7 × 10^3^ and 6.38 × 10^2^ IU/mL, respectively. The study was approved by the Research Ethics Board at the WRH and the University of Windsor. All blood samples were collected with donor consent.

### 2.2 Peripheral Blood Mononuclear Cell Isolation

PBMCs were isolated from peripheral blood samples (collected in heparin tubes) or buffy coats by density gradient centrifugation using Ficoll (Cytiva) following a protocol described previously [39]. Cells were either processed immediately or cryopreserved in 90% fetal bovine serum (FBS; Gibco) and 10% dimethylsulfoxide (DMSO; MP Biochemicals) at a concentration of 2–10 × 10^6^ cells/mL and stored in liquid nitrogen.

### 2.3 CMV Serology

CMV serology was determined using CMV‐IgG enzyme‐linked immunosorbent assay (ELISA) kit (OriGene) following the manufacturer's instructions. A sample of plasma was analyzed to determine the presence or absence of CMV‐IgG in blood according to the cutoff control provided in the kit.

### 2.4 Flow Cytometry Analysis

The following fluorescently labeled antibodies were purchased from BioLegand: PE‐CY7‐CD56 (clone: QA17A16), BV650‐CD3 (clone: OKT3), PE‐NKG2C (clone: S19005E), APC‐NKR‐P1A/CD161 (clone: HP‐3G10), PE/Dazzle 594‐CD161 (clone: HP‐3G10), BV510‐CD16 (clone: 3G8), PE/Dazzle 594‐CD57 (clone: QA17A04), PerCP‐CY5.5‐IFN‐γ (clone: 4S.B3), and PerCP‐CY5.5‐Ki67 (clone: Ki‐67). AF700‐Granzyme B (clone: GB11) was purchased from BD Bioscience. Dead cells were excluded from the analysis using Fixable viability dye (APC‐eF780) purchased from Invitrogen.

Frozen PBMCs were thawed and washed with RPMI medium containing DNase following a previously described protocol [39]. Approximately, 1 × 10^6^ cells were stained with fluorescently labeled antibodies for flow cytometry analysis. PBMCs were treated with Human TruStain FcX Fc receptor blocking solution (Biolegend) following the manufacturer's instructions. Cells were first stained with the fixable viability dye (Invitrogen) in PBS following the manufacturer's recommendations. Cells were then washed with PBS and incubated with fluorescently labeled antibodies at specific concentrations in FACS staining buffer at 4°C for 20–25 min and protected from light. For intracellular staining, cells were washed with FACS buffer after cell surface staining and then fixed and permeabilized using fixation and permeabilization buffers (Invitrogen) following the manufacturer's instructions. Thereafter, cells were incubated at 4°C for 20–25 min with appropriate antibodies diluted in permeabilization buffer and protected from light. Fluorescence‐Minus‐One (FMO) control containing all the fluorochromes of the antibody staining panel, except the one that was being measured, was used to identify and gate cells. Data were acquired using BD LSR Fortessa X20 flow cytometer and analyzed using FlowJo (V10) analysis software.

### 2.5 *In Vitro* Stimulation

Approximately, 1 × 10^6^ frozen PBMCs were thawed and incubated with IL‐2 (1000 U/mL) in complete RPMI‐1640 (Sigma) medium containing 10% FBS (Gibco), 100 U/mL penicillin (Gibco) at 37°C and 5% CO_2_ overnight. Cells were then removed from IL‐2 by centrifugation at 500×*g* for 5 min and treated with Phorbol 12‐myristate 13‐acetate (PMA) (20 ng/mL) and ionomycin (1 µg/mL) or incubated with K562 cells at 1:1 ratio in complete RPMI‐1640 (Sigma) medium in a 96‐well round‐bottom plate for 5 h at 37°C and 5% CO_2_. Brefeldin A (BioLegend), monensin (BioLegend), and BV421‐CD107a (H4A3) antibodies at appropriate dilutions were added to the cells after 30–60 min of incubation. At the end of the 5 h incubation period, cells were stained using fixable viability dye and NK cell surface markers, followed by cell fixation and permeabilization for IFN‐γ intracellular staining as described above.

### 2.6 *In Vitro* Cytokine Treatment and NK Cell Proliferation

For *in vitro* NK cell proliferation, 1 × 10^6^ frozen PBMCs were thawed and incubated with IL‐2 (1000 U/mL) in complete RPMI‐1640 (Sigma) medium containing 10% FBS (Gibco), 100 U/mL penicillin (Gibco) at 37°C and 5% CO_2_ overnight. Cells were then removed from IL‐2 by centrifugation at 500×*g* for 5 min and incubated with IL‐15 (1 ng/mL), or a cocktail of IL‐15 (1 ng/mL), IL‐12 (10 ng/mL), and IL‐18 (50 ng/mL) at 37°C and 5% CO_2_ for 5 days. Cells were then stained using NK cell surface markers and anti‐CD161 (NKR‐P1A) antibody to determine the NKR‐P1A receptor expression followed by fixation and permeabilization for intracellular staining of the nuclear protein Ki‐67 as described above to determine cell proliferation.

### 2.7 Analysis of Apoptotic Cell Death

PBMCs before and after 5 days of cytokine treatment were stained with the fixable viability dye (Invitrogen) in PBS following the manufacturer's recommendations. Cells were then washed with PBS, stained for NK cell surface markers as described above, and Apotracker Green (Biolegend) to stain apoptotic cells following the manufacturer's recommendations.

### 2.8 *In Vitro* Cytokine Treatment and Stimulation of Sorted NK Cells

NK cells were enriched from PBMCs using an NK cell isolation kit (Miltenyi Biotec). Enriched NK cells were stained and CD56^+^NKR‐P1A^+^ cells were sorted. Sorted NK cells were incubated with IL‐15 (1 ng/mL), or a cocktail of IL‐15 (1 ng/mL), IL‐12 (10 ng/mL), and IL‐18 (50 ng/mL) in complete RPMI‐1640 (Sigma) medium in a 96‐well round‐bottom plate at 37°C and 5% CO_2_ for 3 or 5 days. To further stimulate cytokine‐treated cells, cells were harvested, washed, counted, and incubated with K562 cells at a 1:10 (NK:K562) ratio in complete RPMI‐1640 (Sigma) medium in a 96‐well round‐bottom plate for 5 h at 37°C and 5% CO_2_. At days 0, 3, and 5 of cytokine treatment, and after stimulation with K562 cells, NK cells were stained using NK cell surface markers, fixable viability dye, Apotracker Green (BioLegend) for apoptotic cells, and anti‐CD161 (NKR‐P1A) antibody to determine NKR‐P1A receptor expression after exclusion of apoptotic and dead cells.

### 2.9 Processing and Analysis of Publicly Available Single‐Cell RNA Sequence (scRNA‐seq) Data

scRNA‐seq data from Rückert et al. [37] was extracted as a BAM file from Gene Expression Omnibus specified under accession code GSE197037. BAM files were converted to fastq format using 10X Genomics’ bamtofastq (v. 1.3.2). fastq files were processed through Cell Ranger (v8.0.1) and the output files (barcodes.tsv, features.tsv, and matrix.mtx) from CMV^+^ (*n* = 3) and CMV^‒^ (*n* = 2) scRNA‐seq sample folders were imported into RStudio for analysis [40]. Color palettes and quality control metrics were followed as per the Rückert et al. code (https://github.com/timorueckert/Clonal_NK/blob/main/scRNA/01_Main_analysis.R). Cells were filtered on the percentage of mitochondrial reads (including <5% mitochondrial DNA) and the number of reads. Contaminating erythrocytes were filtered out. Count data were imported into Seurat, normalized, and scaled [41]. Data were normalized to 40,000 reads per cell. Preclustering dimensional reduction was performed by computing principal component analysis (PCA) and uniform manifold approximation and projection (UMAP) on the cells using the functions RunPCA and RunUMAP. Clustering via FindNeighbours and FindClusters highlighted a cluster with contaminating innate lymphocytes (mainly ILC2s), which was removed to ensure NK cell purity. The resulting cells were re‐clustered to visualize subpopulations of NK cells based on proliferative capacity and activation state. Data from CMV^+^ samples were merged and integrated via canonical correlation analysis (CCA) to correct for batch effects and were assigned serostatus “CMVpos”. The same was applied to data from CMV^‒^ samples, which were assigned serostatus “CMVneg”. The integrated Seurat objects were normalized, scaled, and clustered. NKR‐P1A/CD161 (+) or (‐) tags were added to both CMVpos and CMVneg Seurat objects based on the expression of the *KLRB1* gene.

For analysis of differentially expressed genes (DEG) on pseudo‐bulk data, count matrices were extracted from CMVpos and CMVneg Seurat objects and down‐sampled based on NKR‐P1A expression (7500 and 1800 cells, respectively) for pseudo‐bulk analysis. Corresponding data frames with log_2_‐foldchanges and *p*‐values of significant DEG were created via the FindMarkers tool. Mitochondrial and ribosomal genes were eliminated from the dataframe, and corresponding volcano and dot plots were generated based on the top 30 most significant genes.

### 2.10 Statistical Analysis

Statistical analysis was performed by unpaired Student's *t*‐test, and one‐way ANOVA followed by Tukey post hoc test where applicable. Differences with *p*‐values of less than or equal to 0.05 were considered statistically significant.

## 3 RESULTS

### 3.1 Accumulation of NKG2C^+^ NK Cells Lacking NKR‐P1A Receptor during CMV Infection

**FIGURE 1:**
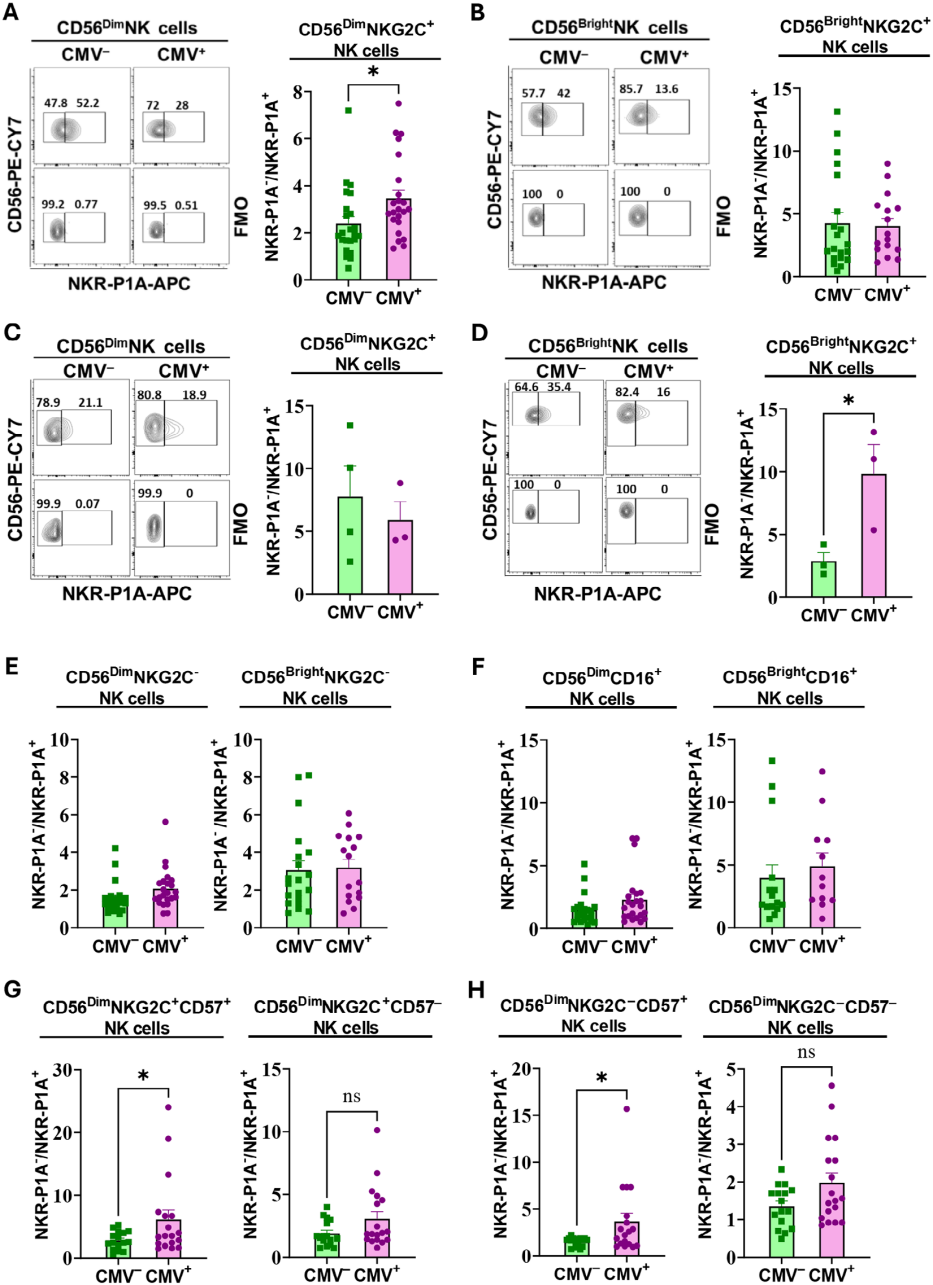
Reduced frequencies of NKR‐P1A^+^ NK cells relative to NKR‐P1A^‒^ NK cells during CMV infection. PBMCs were stained with antibodies against NK cell markers and NKR‐P1A receptor to determine NKR‐P1A expression on NK cells by flow cytometry analysis. (A, B) Representative flow cytometry analysis plots showing percentages of NKR‐P1A^+^ and NKR‐P1A^‒^ cells in CD56^Dim^ (A) and CD56^Bright^ (B) NKG2C^+^ NK cells from CMV seronegative (CMV^‒^) and seropositive (CMV^+^) donors. Fluorescence‐Minus‐One (FMO) control was used to identify positive staining. Bar graphs show mean ± SEM values for the NKR‐P1A^‒^/NKR‐P1A^+^ ratio. Each symbol represents a single donor—CMV^+^ (*n* = 16–23) and CMV^‒^ (*n* = 20–23). (C and D) Representative flow cytometry analysis plots showing percentages of NKR‐P1A^+^ and NKR‐P1A^‒^ cells in CD56^Dim^ (C) and CD56^Bright^ (D) NKG2C^+^ NK cells from HSCT patients with no CMV reactivation (CMV^‒^) and with active CMV infection (CMV^+^). FMO control was used to identify positive staining. Bar graphs show mean ± SEM values for the NKR‐P1A^‒^/NKR‐P1A^+^ ratio. Each symbol represents a single donor—CMV^+^ (*n* = 3) and CMV^‒^ (*n* = 4). (E, F) Bar graphs showing mean ± SEM values for NKR‐P1A^‒^/NKR‐P1A^+^ ratio in CD56^Dim^ and CD56^Bright^ subsets of NKG2C^‒^ (E) and CD16^+^ (F) NK cells from CMV seronegative (CMV^‒^) and seropositive (CMV^+^) donors. Each symbol represents a single donor—CMV^+^ (*n* = 16–23) and CMV^‒^ (*n* = 20–23). (G, H) Bar graphs showing mean ± SEM values for NKR‐P1A^‒^/NKR‐P1A^+^ ratio in CD57^+^ and CD57^‒^ subsets of CD56^Dim^ NKG2C^+^ (G) and CD56^Dim^ NKG2C^‒^ (H) NK cells from CMV seronegative (CMV^‒^) and seropositive (CMV^+^) donors. Each symbol represents a single donor—CMV^+^ (*n* = 18) and CMV^‒^ (*n* = 16). Statistical significance was determined by unpaired Student's *t*‐test.

To determine if the inhibitory NKR‐P1A receptor affected NK cell expansion in CMV‐infected individuals, we analyzed frequencies of NKG2C^+^ NK cells that express or lack expression of NKR‐P1A in PBMCs from CMV‐infected and uninfected donors. We observed a significant increase in the NKR‐P1A^‒^/NKR‐P1A^+^ ratio of NKG2C^+^ NK cells in the CD56^Dim^ (Figure 1A) but not in CD56^Bright^ (Figure 1B) subset of NK cells in CMV seropositive compared with seronegative individuals. Similar analysis of PBMCs from a small number of HSCT patients with active CMV infection demonstrated a significant increase in NKR‐P1A^‒^/NKR‐P1A^+^ ratio in CD56^Bright^ but not CD56^Dim^ NK cells (Figure 1C,D). These results indicate a preferential expansion of NKR‐P1A^‒^ over NKR‐P1A^+^ NK cells during CMV infection. To determine if the expansion of NKR‐P1A^‒^ NK cells is restricted to NKG2C^+^ NK cells, we analyzed NKG2C^‒^ and CD16^+^ NK cells in a similar manner. We found no differences in NKR‐P1A^‒^/NKR‐P1A^+^ ratio in either NKG2C^‒^ (Figure 1E) or CD16^+^ (Figure 1F) subsets of NK cells from CMV seropositive and seronegative individuals, indicating that increased frequencies of cells lacking NKR‐P1A receptor in CMV‐infected individuals are restricted to NKG2C^+^ NK cell subset. Together, these results show that the expansion of NKG2C^+^ NK cells during CMV infection is restricted to the subset lacking NKR‐P1A receptors.

CD57 is a marker for NK cell differentiation and has been proposed to mark adaptive NK cells that expand in response to CMV infection [42]. To determine if NKR‐P1A receptor expression also affected the expansion of adaptive CD57^+^NKG2C^+^ NK cells during CMV infection, we analyzed the frequencies of CD57^+^NKG2C^+^ NK cells that express or lack expression of NKR‐P1A in PBMCs from CMV seropositive and seronegative donors. We observed a statistically significant increase in NKR‐P1A^‒^/NKR‐P1A^+^ ratio in CD57^+^NKG2C^+^ NK cells but not CD57^‒^NKG2C^+^ NK cells from CMV seropositive compared with seronegative individuals (Figure 1G). Interestingly, NKR‐P1A^‒^/NKR‐P1A^+^ ratio was also increased in CD57^+^NKG2C^‒^ NK cells but not CD57^‒^NKG2C^‒^ NK cells from CMV seropositive compared with seronegative individuals, indicating that fewer NKR‐P1A^+^ NK cells in general show a terminally differentiated phenotype in CMV seropositive individuals (Figure 1H). Together, these results demonstrate that fewer NKR‐P1A^+^ compared with NKR‐P1A^‒^ NK cells make up the CMV‐specific adaptive NK cell compartment that expands during CMV infection.

### 3.2 Reduced Proliferation and Activation of NKG2C^+^ NK Cells Expressing NKR‐P1A Receptor during CMV Infection

**FIGURE 2:**
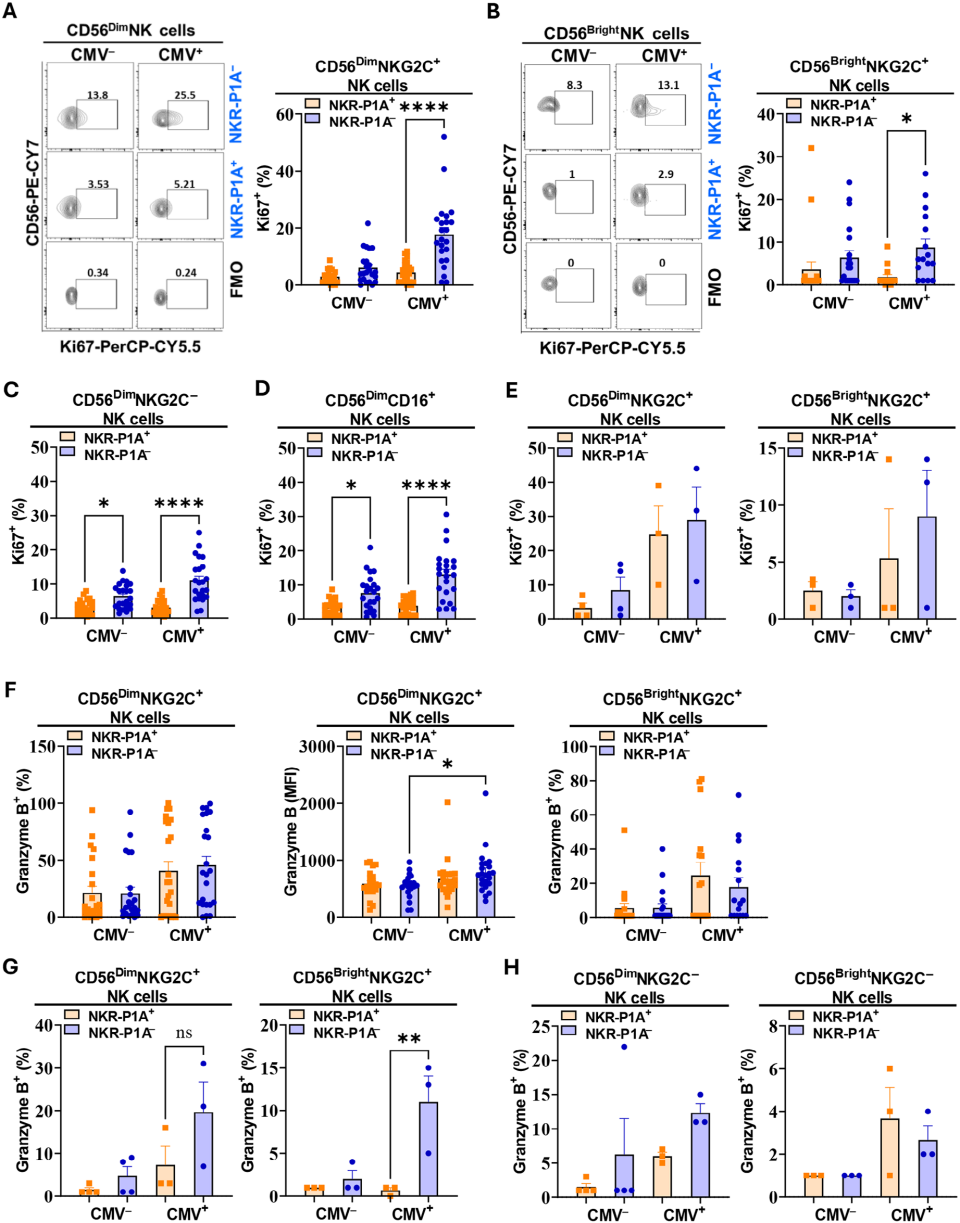
Lower proliferation and activation of NKR‐P1A^+^ NK cells during CMV infection. PBMCs were stained with antibodies for NK cell markers (surface stain) and antibodies for Ki67 and granzyme B (intracellular stain) after cell fixation and permeabilization to determine the expression of Ki67 and granzyme B in NK cells by flow cytometry analysis. (A, B) Representative flow cytometry analysis plots showing percentages of Ki67^+^ cells in NKR‐P1A^+^ and NKR‐P1A^‒^ subsets of CD56^Dim^ (A) and CD56^Bright^ (B) NKG2C^+^ NK cells from CMV seronegative (CMV^‒^) and seropositive (CMV^+^) donors. FMO control was used to identify positive staining. Bar graphs show mean ± SEM values for Ki67^+^ percentages. Each symbol represents a single donor—CMV^‒^ (*n* = 20–23) and CMV^+^ (*n* = 16–23). (C, D) Bar graphs showing mean ± SEM values for Ki67^+^ percentages in NKR‐P1A^+^ and NKR‐P1A^‒^ subsets of NKG2C^‒^ (C) and CD16^+^ (D) CD56^Dim^ NK cells from CMV seronegative (CMV^‒^) and seropositive (CMV^+^) donors. Each symbol represents a single donor—CMV^‒^ (*n* = 23) and CMV^+^ (*n* = 23). (E) Bar graphs showing mean ± SEM values for Ki67^+^ percentages NKR‐P1A^+^ and NKR‐P1A^‒^ subsets of NKG2C^+^ NK cells (CD56^Dim^ and CD56^Bright^) from HSCT patients with no CMV reactivation (CMV^‒^) and with active CMV infection (CMV^+^). Each symbol represents a single donor—CMV^+^ (*n* = 3) and CMV^‒^ (*n* = 4). (F) Bar graphs showing mean ± SEM values for granzyme B^+^ percentages and MFI in NKR‐P1A^+^ and NKR‐P1A^‒^ subsets of CD56^Dim^ NKG2C^+^ NK cells and granzyme B^+^ percentages in CD56^Bright^ NKG2C^+^ NK cells from CMV seronegative (CMV^‒^) and CMV seropositive (CMV^+^) donors. Each symbol represents a single donor—CMV^‒^ (*n* = 20–23) and CMV^+^ (n = 16–23). (G, H) Bar graphs showing mean ± SEM values for granzyme B^+^ percentages in NKR‐P1A^+^ and NKR‐P1A^‒^ subsets of NKG2C^+^ (G) NKG2C^‒^ (H) NK cells (CD56^Dim^ and CD56^Bright^) from HSCT patients with no CMV reactivation (CMV^‒^) and with active CMV infection (CMV^+^). Each symbol represents a single donor—CMV^+^ (*n* = 3) and CMV^‒^ (*n* = 4). Statistical significance was determined by one‐way ANOVA followed by Tukey post hoc test.

The reduced frequency of NK cells expressing NKR‐P1A receptor indicates either downregulation of NKR‐P1A expression in NK cells during CMV infection or increased proliferation of NK cells lacking NKR‐P1A expression resulting in a predominantly NKR‐P1A^‒^ phenotype. To assess NK cell proliferation, we analyzed the expression of Ki67, a nuclear protein highly expressed in proliferating cells, in NKR‐P1A^‒^ and NKR‐P1A^+^ subsets of NK cells from CMV‐infected and uninfected individuals (see gating strategy in Figure S1A). Our results showed significantly lower frequencies of Ki67^+^ cells amongst the NKR‐P1A^+^NKG2C^+^ NK cells compared with the corresponding NKR‐P1A^‒^ cell subset in CMV seropositive individuals (Figure 2A,B). Analysis of Ki67 expression in NKG2C^−^ and CD16^+^ subsets of NK cells also showed reduced steady‐state proliferation in NKR‐P1A^+^ compared with NKR‐P1A^‒^ NK cells (Figure 2C,D), however, unlike in NKG2C^+^ NK cells, these differences were statistically significant in both CMV seropositive and seronegative individuals (Figure 2C,D). In contrast, analysis of Ki67 expression in NK cells from HSCT patients showed similar levels of proliferation in NKR‐P1A^+^ and NKR‐P1A^‒^ NK cells during active CMV infection (Figure 2E). Together, these results demonstrate higher intrinsic steady‐state proliferation capacity in NK cells lacking expression of NKR‐P1A receptor compared with those expressing NKR‐P1A. Importantly, proliferation of CMV‐specific NKG2C^+^ NK cells in CMV seropositive individuals is restricted to the subset of cells lacking NKR‐P1A receptor.

To further assess the activation status of NK cells expressing NKR‐P1A receptor during CMV infection, we compared the expression of granzyme B in NKR‐P1A^+^ and NKR‐P1A^‒^ NK cells (see gating strategy in Figure S1B). Our results showed no differences in the frequencies of granzyme B^+^ cells in the NKR‐P1A^+^ and NKR‐P1A^‒^ subsets of NK cells from CMV seropositive individuals (Figure 2F). A comparison of the mean fluorescence intensity (MFI) of granzyme B staining in NK cells also did not show significant differences between NKR‐P1A^+^ and NKR‐P1A^‒^ subsets; however, there was a small but statistically significant increase in granzyme B MFI values in NKR‐P1A^‒^ NK cell subset from CMV seropositive compared with CMV seronegative individuals (Figure 2F). In HSCT patients with active CMV infection, NKR‐P1A^‒^ NK cells showed significantly higher frequencies of granzyme B^+^ cells compared with NKR‐P1A^+^ NK cells (Figure 2G). This was also specific to the NKG2C^+^ subset of NK cells since no statistically significant differences in the frequencies of granzyme B^+^ cells in NKR‐P1A^+^ and NKR‐P1A^‒^ subsets of NKG2C^‒^ NK cells were observed in HSCT patients with active CMV infection (Figure 2H). Together, these results demonstrate that NK cells expressing the NKR‐P1A receptor exhibit a less active phenotype and reduced proliferation compared with those lacking NKR‐P1A receptor expression during CMV infection.

### 3.3 NKR‐P1A Receptor Expression Is Retained on NK Cell after *in Vitro* Stimulation

**FIGURE 3:**
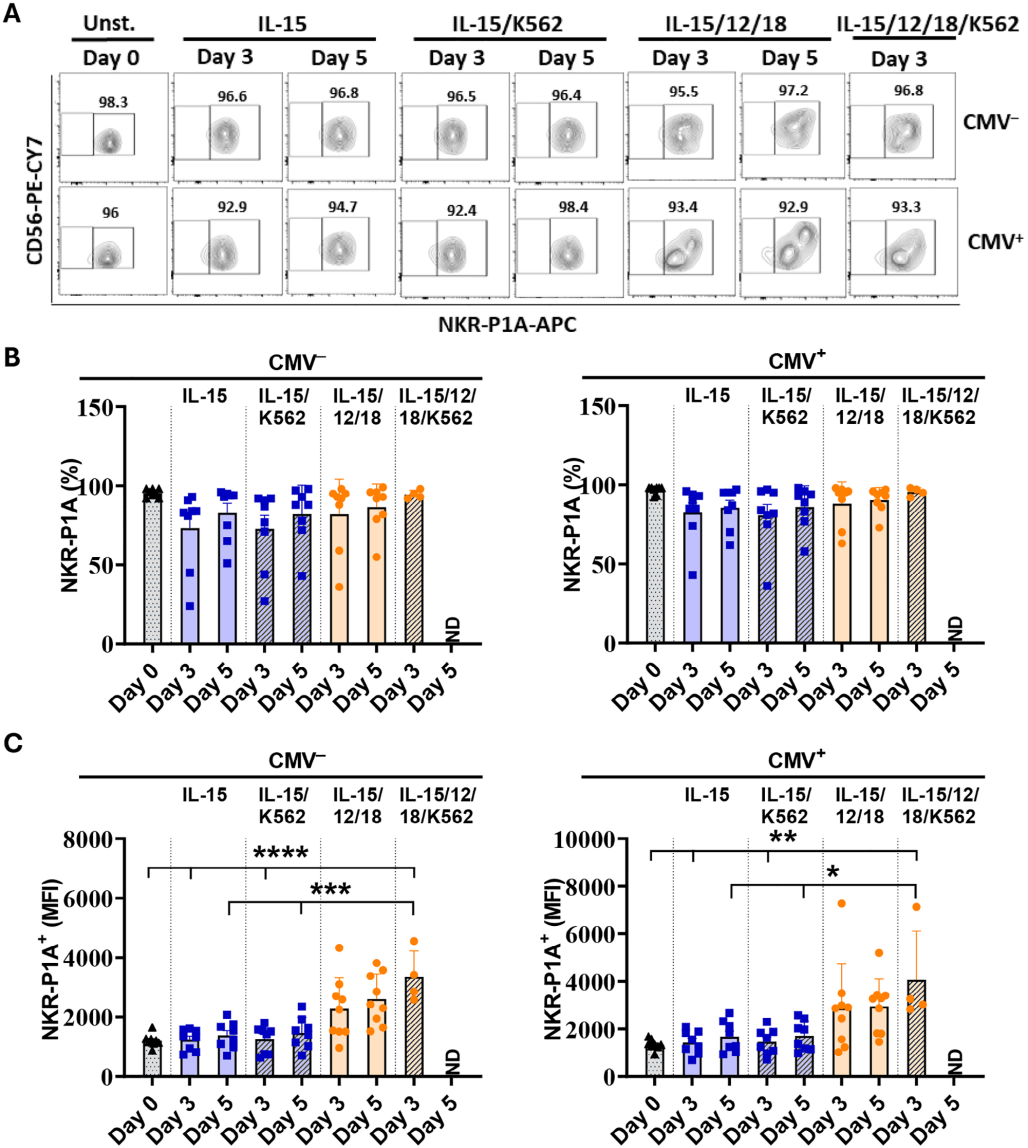
NKR‐P1A receptor expression is retained on activated NK cells. NKR‐P1A^+^ NK cells were sorted from PBMCs and treated with indicated cytokines for 3 or 5 days followed by stimulation with K562 cells for 5 h. Cells were stained for flow cytometry analysis and NKR‐P1A receptor expression was assessed on live NK cells after exclusion of apoptotic and dead cells. (A) Representative flow cytometry plots showing NKR‐P1A receptor expression on sorted NK cells in unstimulated (Day 0), and those treated with IL‐15, or a combination of IL‐15, IL‐12, and IL‐18, and cytokine treatment followed by stimulation with K562 tumor cells. Percent values for NKR‐P1A^+^ cells are indicated. (B, C) Bar graphs showing mean ± SEM values for percentage (B) and MFI values (C) for NKR‐P1A staining in sorted NK cells at day 0 (unstimulated), after treatment with IL‐15 or IL15, IL‐12, and IL‐18 for 3 and 5 days, and after K562 tumor cell stimulation following cytokine treatment. Each symbol represents a single donor—CMV^+^ (*n* = 8) and CMV^‒^ (*n* = 8) for all stimulations except IL‐15, IL‐12, IL‐18, and K562 stimulation on Day 3 (*n* = 4 for CMV^+^ and CMV^‒^ each). Statistical significance was determined by one‐way ANOVA followed by Tukey post hoc test.

To determine if the NK cell activation program can result in the downregulation of NKR‐P1A receptor expression in NK cells, we treated sorted NKR‐P1A^+^ NK cells from CMV‐infected and uninfected individuals *in vitro* with IL‐15 alone or a combination of IL‐12, IL‐15, and IL‐18 for 3 or 5 days. On days 3 and 5, a portion of the cells were further stimulated with K562 tumor cells for 5 h. After the exclusion of apoptotic and dead cells, we analyzed NKR‐P1A receptor expression on CD56^+^ NK cells. Neither the percentage of NK cells expressing the NKR‐P1A receptor, nor the MFI value for NKR‐P1A receptor staining on NK cells was significantly reduced when NK cells were stimulated with IL‐15 or IL‐15 followed by K562 cells (Figure 3A–C). Similarly, the percentage of NK cells expressing NKR‐P1A receptor remained unaffected, but NKR‐P1A receptor expression was upregulated (significantly higher MFI values) when sorted NK cells were stimulated with a combination of IL‐12, IL‐15, and IL‐18 with and without K562 stimulation compared with the unstimulated (day 0) and IL‐15 stimulated cells (Figure 3A–C). IL‐12 has previously been shown to induce upregulation of NKR‐P1A receptor expression in NK cells [43]. Together, these results show that NKR‐P1A receptor expression is not lost during NK cell activation.

### 3.4 *In Vitro* Cytokine Treatment Restores NKR‐P1A^+^ NK Cell Proliferation but Not Cell Frequency

**FIGURE 4:**
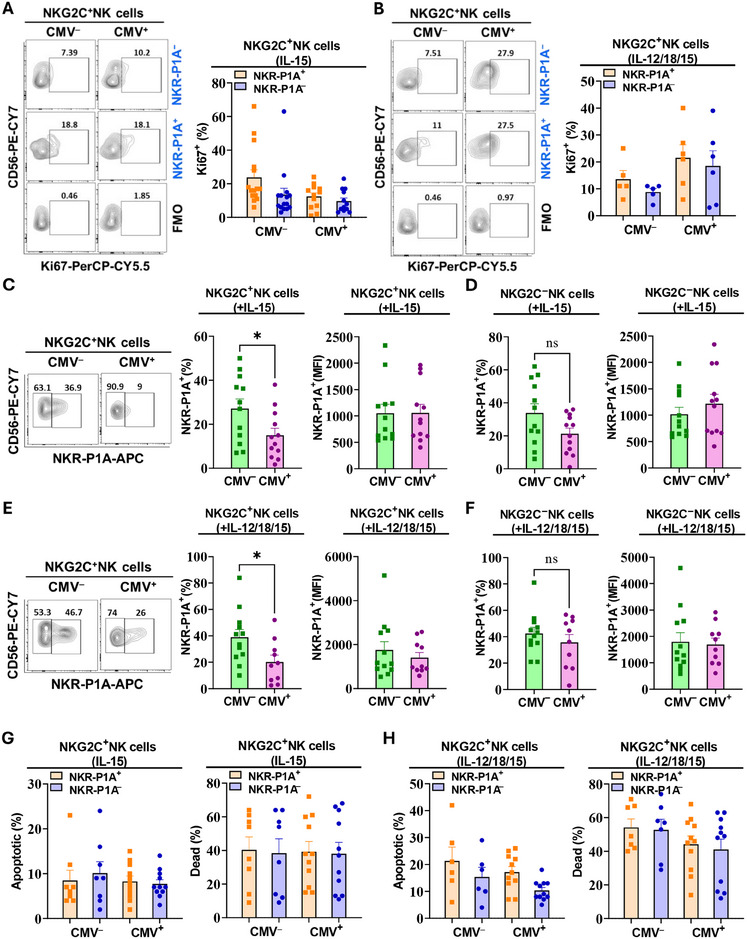
Restoration of NKR‐P1A^+^ NK cell proliferation but not frequency by cytokines *in vitro*. PBMCs were treated with IL‐15 alone or in combination with IL‐12 and IL‐18 (IL‐12/18/15) for 5 days. Cells were then stained for flow cytometry analysis of NKR‐P1A and Ki67 expression, and apoptosis in NK cells. (A, B) Representative flow cytometry analysis plots showing percentages of Ki67^+^ cells in NKR‐P1A^+^ and NKR‐P1A^‒^ subsets of NKG2C^+^ NK cells from CMV seronegative (CMV^‒^) and seropositive (CMV^+^) donors treated with IL‐15 (A) or IL‐12/18/15 (B). FMO control was used to identify positive staining. Bar graphs show mean ± SEM values for Ki67^+^ percentages. Each symbol represents a single donor—CMV^‒^ (*n* = 5–15) and CMV^+^ (*n* = 6–12). (C, D) A representative flow cytometry analysis plot showing NKR‐P1A^+^ and NKR‐P1A^‒^ percentages in NKG2C^+^ NK cells (C) from CMV seronegative (CMV^‒^) and seropositive (CMV^+^) donors treated with IL‐15. Bar graphs show mean ± SEM values for NKR‐P1A^+^ percentage and mean fluorescence intensity (MFI) values for NKR‐P1A staining in NKG2C^+^ (C) and NKG2C^‒^ (D) NK cells. Each symbol represents a single donor—CMV^‒^ (*n* = 12) and CMV^+^ (*n* = 12). (E, F) A representative flow cytometry analysis plot showing NKR‐P1A^+^ and NKR‐P1A^‒^ percentages in NKG2C^+^ NK cells (E) from CMV seronegative (CMV^‒^) and seropositive (CMV^+^) donors treated with IL‐12/18/15. Bar graphs show mean ± SEM values for NKR‐P1A^+^ percentage and MFI values for NKR‐P1A staining in NKG2C^+^ (E) and NKG2C^‒^ (F) NK cells. Each symbol represents a single donor—CMV^‒^ (*n* = 12) and CMV^+^ (*n* = 10). Statistical significance was determined by Student's *t*‐test. (G, H) Bar graphs show mean ± SEM values for the percentages of apoptotic and dead cells in NKR‐P1A^+^ and NKR‐P1A^‒^ subsets of NKG2C^+^ NK cells from CMV seronegative (CMV^‒^) and seropositive (CMV^+^) donors treated with IL‐15 (G) or IL‐12/18/15 (H). Each symbol represents a single donor—CMV^‒^ (*n* = 6–8) and CMV^+^ (*n* = 11). Statistical significance was determined by one‐way ANOVA followed by Tukey post hoc test.

To determine the inherent proliferative capacity of NKR‐P1A^+^ NK cells and whether their proliferation can be restored, we treated PBMCs from CMV‐infected and uninfected individuals *in vitro* with IL‐15 alone or a combination of IL‐12, IL‐15, and IL‐18 for 5 days. IL‐15 triggered proliferation in NKG2C^+^ NK cells from CMV seropositive and seronegative individuals to the same level irrespective of their NKR‐P1A receptor expression status (Figure 4A). A combination of IL‐12, IL‐15, and IL‐18 induced a more robust proliferation in NKG2C^+^ NK cells from CMV seropositive compared with seronegative individuals irrespective of NKR‐P1A receptor expression (Figure 4B). In our assays, treatment of NK cells with IL‐15 alone and in combination with IL‐12, and IL‐18 for 5 days increased frequencies of NKR‐P1A^+^ cells in NKG2C^+^ NK cell subsets from CMV seronegative individuals significantly more than CMV seropositive individuals (Figure 4C,E). NKR‐P1A^+^ cell frequencies in NKG2C^‒^ NK cells were not significantly different in CMV seronegative and seropositive individuals after *in vitro* treatment with cytokines (Figure 4D,F). To determine whether NKR‐P1A receptor expression level on a per‐cell basis was affected by CMV infection, we analyzed MFI values for NKR‐P1A staining in NK cells from CMV seronegative and seropositive individuals treated with cytokines *in vitro*. There were no differences in the MFI values for NKR‐P1A staining in NK cells from CMV seronegative and seropositive individuals, indicating that NKR‐P1A expression in neither NKG2C^+^ (Figure 4C,E) nor NKG2C^‒^ NK cells (Figure 4D,F) were affected by CMV infection. Furthermore, to determine if CMV infection differentially affected NKR‐P1A^+^ NK cell survival, leading to reduced frequencies of NKR‐P1A^+^ NK cells from CMV seropositive individuals after *in vitro* cytokine treatment, we assessed apoptotic cell death in NK cells. Our analysis showed similar levels of apoptotic cell death in NK cells from CMV seronegative and seropositive individuals treated with cytokines *in vitro* irrespective of their NKR‐P1A receptor expression status (Figure 4G,H). Together, these results demonstrate that NKR‐P1A^+^ and NKR‐P1A^‒^ NK cells from CMV‐infected individuals are responsive toward cytokines and proliferate equally well *in vitro*, and that cytokine‐induced NKR‐P1A receptor upregulation is not affected by CMV infection. However, frequencies of NKR‐P1A^+^ NK cells remained lower in CMV seropositive compared with seronegative individuals after *in vitro* stimulation, perhaps due to their initial lower frequencies (Figure 1A).

### 3.5 Both NKR‐P1A^+^ and NKR‐P1A^‒^ NK Cells Retain the Capacity to Respond to Stimuli *in Vitro*

**FIGURE 5:**
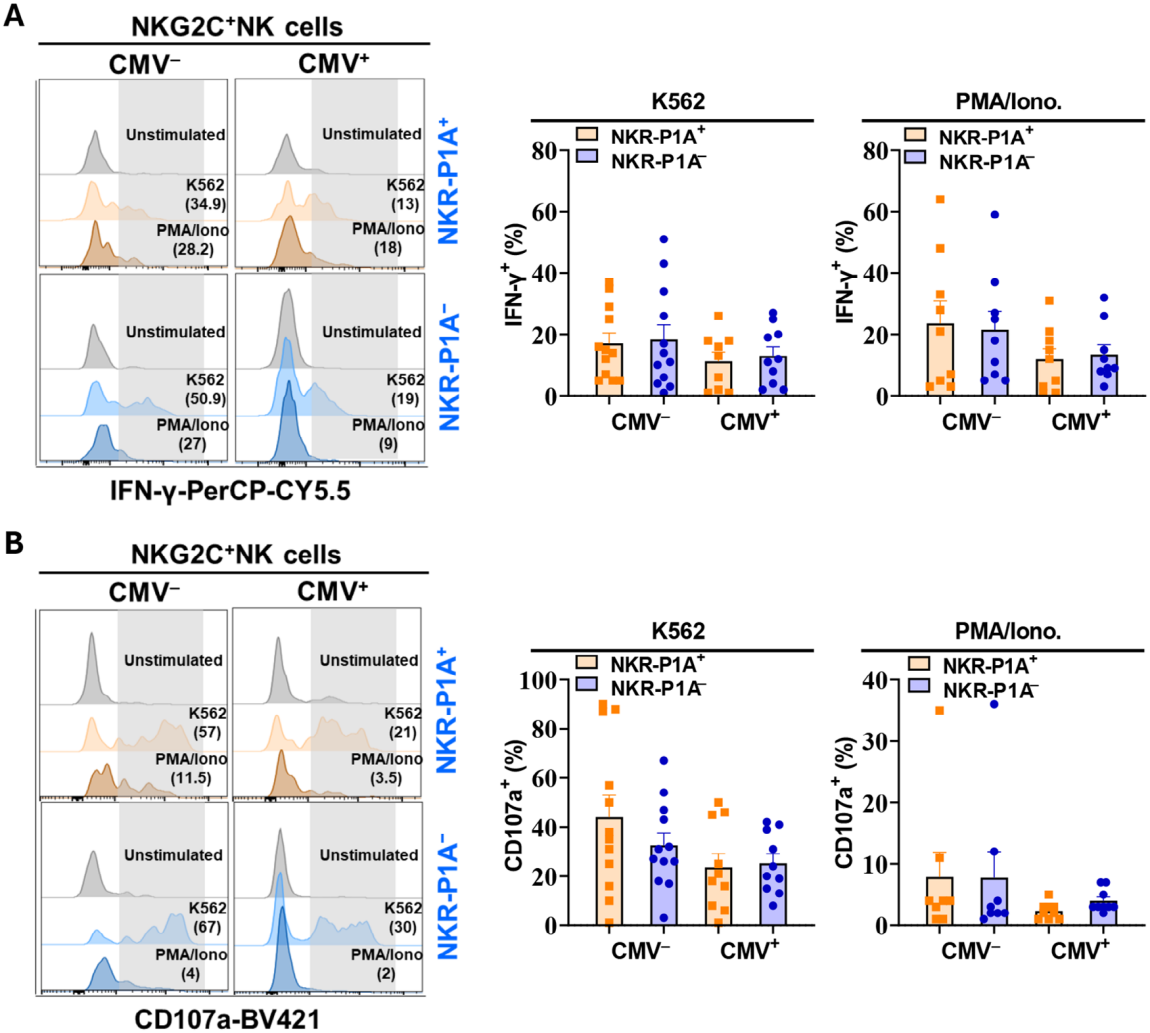
NKR‐P1A^+^ and NKR‐P1A^‒^ NK cells are functionally competent in vitro. PBMCs were incubated with K562 cells or PMA and Ionomycin (PMA/Iono) for 5 h in the presence of inhibitors of the Golgi apparatus. Cells were then stained for flow cytometry analysis of intracellular IFN‐γ and cell degranulation (CD107a) in NK cells. (A, B) Representative overlay histogram plots showing percentages (in parentheses) of IFN‐γ^+^ (A) and CD107a^+^ (B) cells (shaded box) in NKR‐P1A^+^ and NKR‐P1A^‒^ subsets of NKG2C^+^ NK cells from CMV seronegative (CMV^‒^) and seropositive (CMV^+^) donors left untreated (grey histogram) or incubated with K562 cell or PMA/Iono (colored histograms). Bar graphs show mean ± SEM values for IFN‐γ^+^ (A) and CD107a^+^ (B) percentages. Each symbol represents a single donor—CMV^‒^ (*n* = 8–12) and CMV^+^ (*n* = 9–10).

To determine if the phenotypic activation and proliferative differences between NKR‐P1A^+^ and NKR‐P1A^‒^ subsets of NKG2C^+^ NK cells in CMV seropositive individuals may also influence their response to stimuli, we assessed IFN‐γ production (IFN‐γ^+^) and degranulation (CD107a^+^) in NK cells stimulated with PMA and Ionomycin (PMA/I) or K562 tumor cells *in vitro*. We found that although NKG2C^+^ NK cell functions were slightly decreased in CMV seropositive compared with seronegative individuals, both NKR‐P1A^+^ and NKR‐P1A^‒^ NK cells from CMV seropositive individuals showed the same levels of IFN‐γ production (IFN‐γ^+^) (Figure 5A) and degranulation (CD107a^+^) (Figure 5B) when stimulated with PMA/I or K562 tumor cells. These results demonstrate that both NKR‐P1A^+^ and NKR‐P1A^‒^ NK cells in healthy CMV seropositive individuals show similar functionality when stimulated *in vitro*, and that their response to external stimuli is not differentially affected by CMV infection.

### 3.6 CMV Infection Induces Distinct Transcriptional Programs in NKR‐P1A^+^ versus NKR‐P1A^‒^ NK Cells

**FIGURE 6:**
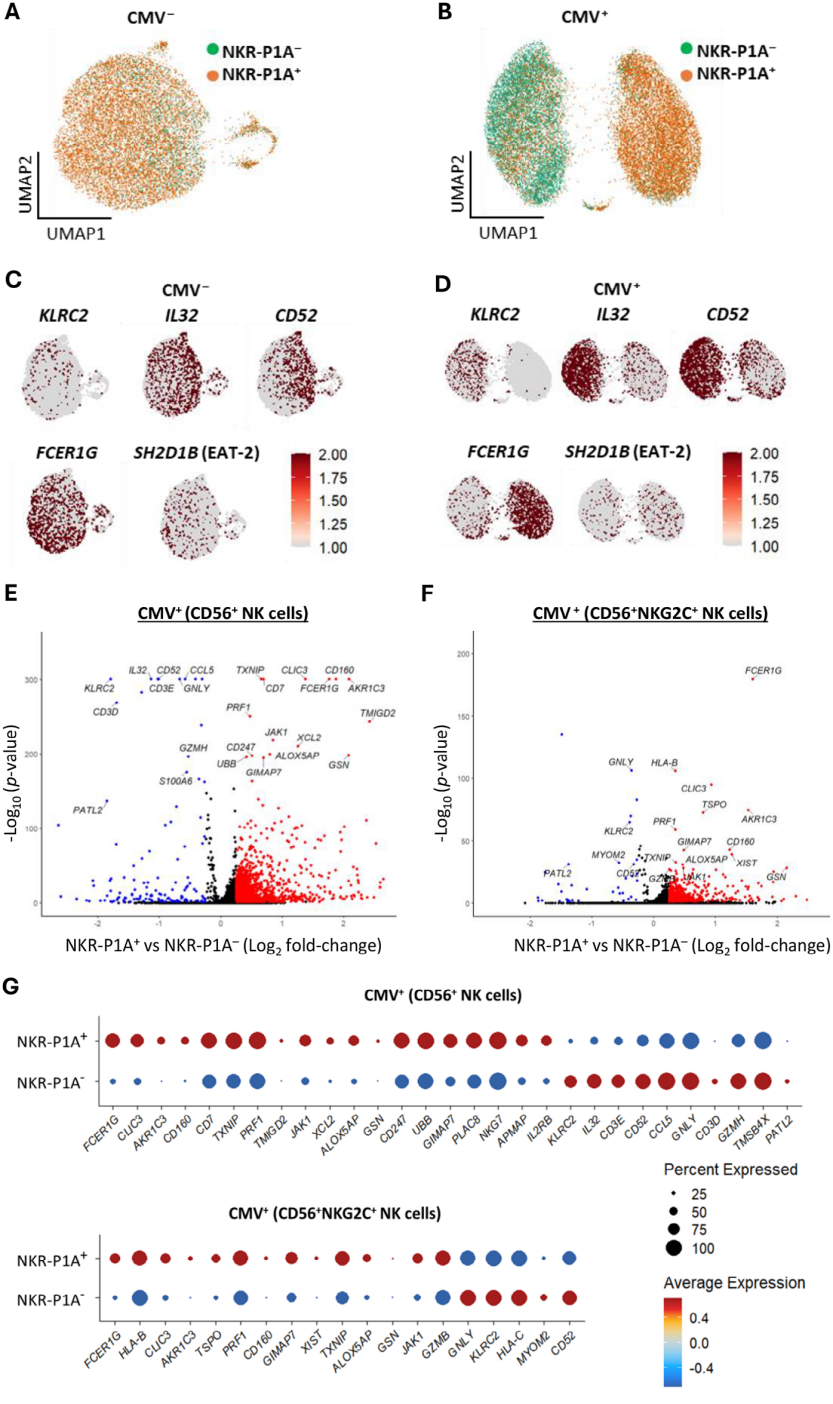
Distinct transcriptome signatures of NKR‐P1A^+^ and NKR‐P1A^‒^ NK cells during CMV infection. Analysis of publicly available NK cell single‐cell RNA sequence (scRNA‐seq) data from CMV seronegative (CMV^‒^) and seropositive (CMV^+^) donors. (A, B) Distribution of NKR‐P1A^+^ and NKR‐P1A^‒^ NK cell populations in UMAP embedding of NK cells from CMV^‒^ (A) and CMV^+^ (B) donors. (C and D) Representative expression of genes in NK cells from CMV^‒^ (C) and CMV^+^ (D) donors. (E and F) Differentially expressed genes between NKR‐P1A^+^ and NKR‐P1A^‒^ subsets of CD56^+^ NK cells (E) and adaptive CD56^+^NKG2C^+^ NK cells (F) from CMV^+^ donors. (G) Gene expression and detection frequencies of differentially expressed genes between NKR‐P1A^+^ and NKR‐P1A^‒^ subsets of CD56^+^ NK cells (E) and adaptive CD56^+^NKG2C^+^ NK cells (F) from CMV^+^ donors.

In a study by Rückert et al. using a single‐cell multi‐omic approach to analyze gene expression (scRNA‐seq) and gene accessibility (scATAC‐seq) profiles in adaptive NK cells, *KLRB1* gene, which encodes for NKR‐P1A receptor, was one of the genes whose expression was markedly downregulated in CMV seropositive individuals [37]. This observation corroborates our data showing expansion of NKG2C^+^ NK cells lacking NKR‐P1A receptor expression in CMV‐infected individuals (Figure 1A). Therefore, to gain insight into the impact of NKR‐P1A receptor expression on NK cell responses during CMV infection, we analyzed the scRNA‐seq dataset from the Rückert et al. study to directly compare transcriptional profiles and distribution of NKR‐P1A^+^ and NKR‐P1A^‒^ NK cells within NK cell clusters in CMV seronegative and CMV seropositive individuals. Analysis of scRNA‐seq datasets separated by CMV serostatus showed that NKR‐P1A^+^ cells were randomly dispersed among NKR‐P1A^‒^ cells in CMV seronegative individuals (Figure 6A). In contrast, NKR‐P1A^+^ and NKR‐P1A^‒^ cells were segregated into separate NK cell clusters in CMV seropositive individuals (Figure 6B). NK cells lacking expression of *KLRB1* gene (NKR‐P1A^‒^) were predominantly present in the NK cell cluster which expressed *KLRC2* gene (NKG2C^+^) (Figure 6D) consistent with the Rückert et al. study [37]. The NKR‐P1A^‒^‐dominated NK cell cluster in CMV seropositive individuals also showed high expression of *IL32* and *CD52*, and low expression of *FCER1G* and *SH2D1B* (EAT‐2) genes (Figure 6D), which are characteristics of adaptive NK cells [38, 44]. In CMV seronegative individuals, these genes were mostly distributed throughout the NK cell clusters (Figure 6C). Expression of *B3GAT1* gene encoding beta‐1,3‐glucuronyltransferase 1, which is required for the synthesis of CD57 carbohydrate epitope associated with adaptive NK cells [42], as well as *SYK* and *ZBTB16* (encoding PLZF) gene whose expression is downregulated or absent in adaptive NK cells [38], were not detected in the scRNA‐seq datasets. Therefore, we analyzed gene expression differences in NKR‐P1A^+^ and NKR‐P1A^‒^ subsets of total CD56^+^ NK cells and adaptive CD56^+^NKG2C^+^ NK cells. Gene expression profiles of NKR‐P1A^+^ and NKR‐P1A^‒^ NK cells in CMV seronegative individuals were largely similar, and any differences in gene expression were minor (Figure S2). In contrast, NKR‐P1A^+^ and NKR‐P1A^‒^ subsets in total NK cells and adaptive NKG2C^+^ NK cells in CMV seropositive individuals exhibited vastly different transcriptomes, highlighting the differential impact of CMV infection on NK cells expressing NKR‐P1A receptor (Figure 6E,F). The highly expressed genes in NKR‐P1A^+^ NK cells from CMV seropositive individuals included genes that encode effector molecules (*PRF1 and GZMB*), cytokine and chemokine responses (*IL2RB, JAK1, XCL2*), and markers associated with NK cell maturity (*FCER1G*, *CLIC3*, *CD160*, *CD7*, *CD247*, *NKG7*) (Figure 6G) [44, 45]. Genes that were highly expressed in NKR‐P1A^‒^ NK cells in CMV seropositive individuals included several genes associated with adaptive NK cells, encoding cell surface proteins and receptors (*KLRC2*, *CD52*), cytokines and chemokines (*IL32*, *CCL5*), CD3 protein chains (*CD3E*) and cytotoxic molecules (G*NLY*, *GZMH*) (Figure 6G) [38, 45]. Together, these results demonstrate that CMV infection differentially impacts NK cells based on their expression of the NKR‐P1A receptor, leading to highly divergent gene expression profiles and responses in NKR‐P1A^+^ versus NKR‐P1A^‒^ NK cells.

## 4 Discussion

CMV causes a latent infection in healthy individuals but a severe and fatal infection in immunocompromised individuals and newborns if not treated. In healthy individuals, NK cell functions are critical for immunity against CMV infection. NK cell functions are regulated by an array of germline‐encoded activating and inhibitory receptors, whose involvement in immunity against CMV infection is highlighted by stable imprints of CMV infection on NK cell receptor repertoire. CMV infection promotes clonal expansion of NK cells expressing the activating receptors NKG2C and members of killer cell immunoglobulin‐like receptors (KIR) (KIR2DS2, KIR2DS4, KIR3DS1), and a bias for self‐specific inhibitory KIRs (educated/licensed NK cell subsets) [18, 46]. A loss of expression of NKR‐P1A (CD161), an inhibitory NK cell receptor, has been observed during CMV infection in several studies [36, 37, 46]. Our current study shows reduced activation and proliferation of NKG2C^+^ NK cells expressing NKR‐P1A receptor compared with those lacking NKR‐P1A in CMV seropositive individuals, which could contribute to the accumulation of NKR‐P1A^‒^ NK cells during CMV infection. NK cells possess adaptive immune features such as expansion of specific NK cell subsets and long‐term immunological memory responses [2]. Our study also shows that the adaptive NKG2C^+^ NK cell compartment predominantly lacks NKR‐P1A receptor expression in CMV seropositive individuals. Despite the reduced proliferation of NKR‐P1A^+^ NK cells in CMV seropositive individuals, they retained the capacity to respond to *in vitro* stimulation comparable to NKR‐P1A^‒^ NK cells.

The NKR‐P1 receptor family in humans consists of C‐type lectin‐like inhibitory NKR‐P1A and activating NKp65 and NKp80 receptors [47]. NKR‐P1A is expressed on a subset of NK cells and its interaction with LLT1 inhibits NK cell cytotoxicity [48]. We show that the NKR‐P1A receptor expression status of NK cells is associated with diverse transcriptional programs induced during CMV infection in NK cells. CMV infection drives clonal expansion of NKG2C^+^ NK cells lacking NKR‐P1A receptor expression. CMV infection does not appear to affect the level of expression of NKR‐P1A receptor since cytokine‐induced upregulation of NKR‐P1A receptor expression was unaffected in NK cells from CMV seropositive individuals. NK cell activation also does not downregulate NKR‐P1A receptor expression, since NKR‐P1A receptor expression was retained in sorted NK cells stimulated with cytokines and K562 tumor cells *in vitro*. Rather, CMV infection appears to differentially affect activation and proliferation specifically in NKG2C^+^ NK cells in a manner that is inversely associated with NKR‐P1A receptor expression. NKR‐P1A^‒^ NK cells in general appear to have a higher homeostatic proliferation compared with NKR‐P1A^+^ NK cells in CMV seronegative individuals. However, in the NKG2C^+^ NK cell compartment, which expands during CMV infection, proliferating cells exclusively lack NKR‐P1A receptor expression. NKG2C^+^ NK cells that lack NKR‐P1A receptor expression also exhibit higher activation frequency compared with NKR‐P1A^+^ NK cells during active CMV infection, as our analysis of granzyme B expression in NK cells from HSCT patients showed. Consequently, the higher activation and proliferation of NKR‐P1A^‒^ compared with NKR‐P1A^+^ NK cells during CMV infection can lead to their increased frequencies observed in the peripheral blood from CMV seropositive individuals. While *in vitro* stimulation of NK cells did not downregulate NKR‐P1A receptor expression, we have not been able to rule out whether adaptive programming in NKG2C^+^ NK cells in CMV seropositive individuals downregulates NKR‐P1A receptor expression. Also, whether the increased frequencies of NK cells lacking NKR‐P1A receptor expression has a role in defense against CMV infection or is merely a byproduct of the infection remains to be determined.

Differential activation and proliferation of NK cells with respect to NKR‐P1A receptor expression may also impact NK cell maturation and acquisition of memory phenotype in response to CMV infection. Indeed, observations in the current and previous studies [49] have shown lower frequencies of NKR‐P1A^+^ cells within the CD57^+^NKG2C^+^ NK cell population, which has been proposed to represent adaptive NK cells in CMV seropositive individuals [42]. In contrast, NKR‐P1A^‒^ NK cells predominantly possess transcriptome signatures that are associated with adaptive NK cells in CMV seropositive individuals. The NK cell compartment in an individual is highly dynamic. Changes in NK cell compartment during virus infection include activation and proliferation, acquisition of tissue residency in affected tissues, changes in receptor expression, maturation, terminal differentiation, and appearance of adaptive memory NK cells, which exhibit properties such as clonal expansion, persistence, and recall responses [50]. Clonal expansion of adaptive CD57^+^NKG2C^+^ NK cells observed in various virus infections in humans has been linked to an underlying CMV infection [51]. Since clonal expansion is a hallmark feature of adaptive memory NK cells, reduced activation and proliferation of NKG2C^+^ NK cells that correlate with NKR‐P1A receptor expression may lead to lower frequencies of NKR‐P1A^+^ cells among CD57^+^NKG2C^+^ NK cells that possess transcriptome features associated with adaptive NK cells in CMV seropositive individuals.

Paired activating and inhibitory immune receptors encoded within the same gene cluster have evolved in response to pathogen infections [52]. Paired NK cell receptors play important roles in immunity against CMV infection, as evident by the opposing roles of the inhibitory NKG2A and activating NKG2C receptors in the recognition of the leader peptide from viral protein UL40 presented in complex with HLA‐E (HLA‐E:pUL40) [20, 22]. Engagement of inhibitory NK cell receptors by viral immunoevasins in both humans and rodents inhibits NK cell responses against CMV infection [20, 24, 27]. The activating members of the paired receptors have evolved to counteract this immunoevasive strategy by recognizing viral immunoevasins, resulting in NK cell activation [22, 24, 27]. The inhibitory NKR‐P1B receptor in both rats and mice is a target of viral immunoevasins by rat CMV (RCMV) and MCMV, respectively [27, 53]. RCMV genome encodes a C‐type lectin‐like protein designated RCTL, which was found to interact with rat NKR‐P1B receptors to inhibit NK cell responses in RCMV‐infected rats. Notably, the activating rat NKR‐P1A receptor also recognizes RCTL, albeit weakly, suggesting the evolution of a counter‐recognition mechanism in rats [53]. Similarly, MCMV genome‐encoded m12 protein acts as a viral immunoevasin to inhibit NK cell responses by interacting with the inhibitory mouse NKR‐P1B receptor. However, the MCMV m12 protein is also recognized by activating mouse NKR‐P1C and NKR‐P1A receptors to counteract NK cell inhibition [27]. Human NKR‐P1A is considered most closely related to rodent NKR‐P1 receptors. While our current study shows a correlation between reduced proliferation in adaptive NKG2C^+^ NK cells and NKR‐P1A receptor expression in CMV seropositive individuals, it does not reveal that NKR‐P1A is a negative regulator of NK cell responses during CMV infection. The impact of NKR‐P1A receptor interactions with its endogenous ligand LLT1 or an unknown viral immunoevasin, as well as the expansion of NK cells lacking NKR‐P1A receptor expression on anti‐CMV immune responses, remains to be determined.

In summary, the relative accumulation of clonally expanded NKG2C^+^ NK cells lacking NKR‐P1A receptor expression during CMV infection correlates with reduced activation and proliferation of NKR‐P1A^+^ NK cells, resulting in under‐representation of NKR‐P1A^+^ NK cells in the adaptive NK cell compartment. This study further highlights the impact of CMV infection in shaping NK cell receptor repertoire.

## Author Contributions

Mohamad Basem Alkassab conducted experiments and analyzed data. Fareeha Ajmal Shaikh analyzed data. Mir Munir A. Rahim and Caroline Hamm conceived the project and supervised the research. Mohamad Basem Alkassab, Fareeha Ajmal Shaikh, Caroline Hamm, and Mir Munir A. Rahim wrote the paper.

## Ethics Statement

The study was approved by the Research Ethics Board at the Windsor Regional Hospital and the University of Windsor. All blood samples were collected with donor consent.

## Conflicts of Interest

The authors declare no conflicts of interest.

## Supporting information



Supporting Information

## Data Availability

Publicly available RNA sequence datasets are available in the Gene Expression Omnibus under the accession code GSE197037. These data were derived from the following resources available in the public domain: https://doi.org/10.1038/s41590‐022‐01327‐7
