# Supplementary material for: Adaptive NKG2C+ NK cells in cytomegalovirus seropositive individuals predominantly lack NKR‐P1A receptor expression
Source: Eur J Immunol. 2025 May 27;55(5):e202451562. doi: 10.1002/eji.202451562 (PMC12117011; doi:10.1002/eji.202451562)
Supplement: Supplementary file 1 — Supporting Information [file EJI-55-e202451562-s001.pdf]

Supplemental Figure 1

A

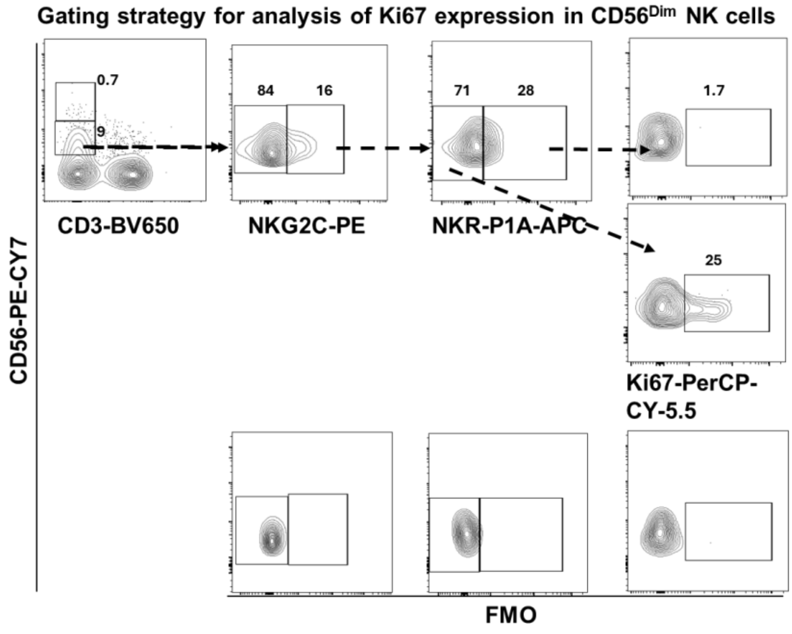

B

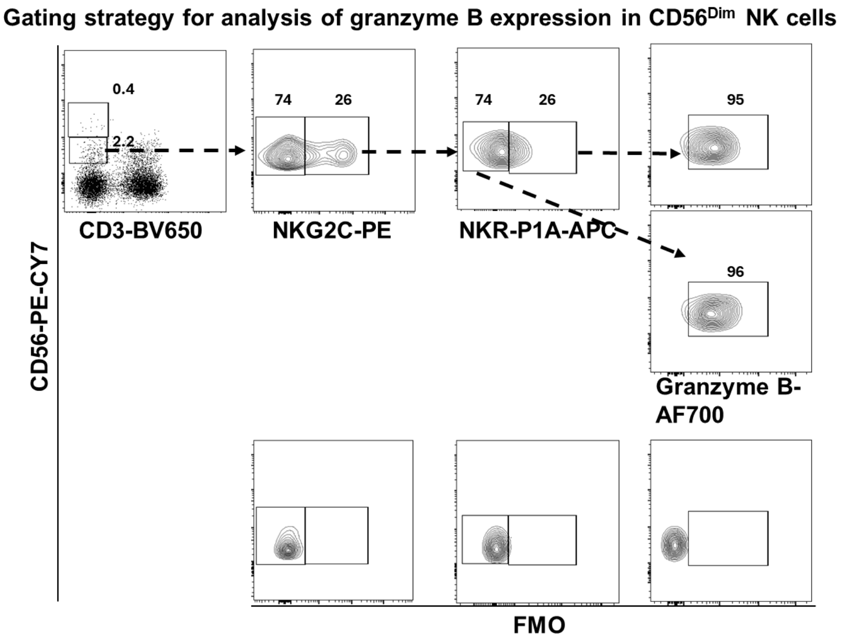

Supplemental Figure 1. Gating strategy for analysis of Ki67 and granzyme B expression in CD56Dim NK cells. NK cells were stained with antibodies against NK cell markers, NKG2C, NKR-P1A, Ki67, and granzyme B for flow cytometry analysis. **A** and **B**) Representative flow cytometry plots for antibody staining and fluorescence minus one (FMO) control show Ki67 (**A**) and granzyme B (**B**) expression in NKR-P1A+ and NKR-P1A- subsets of $CD56^{Dim}NKG2C^{+}$ NK cells.

Supplemental Figure 2

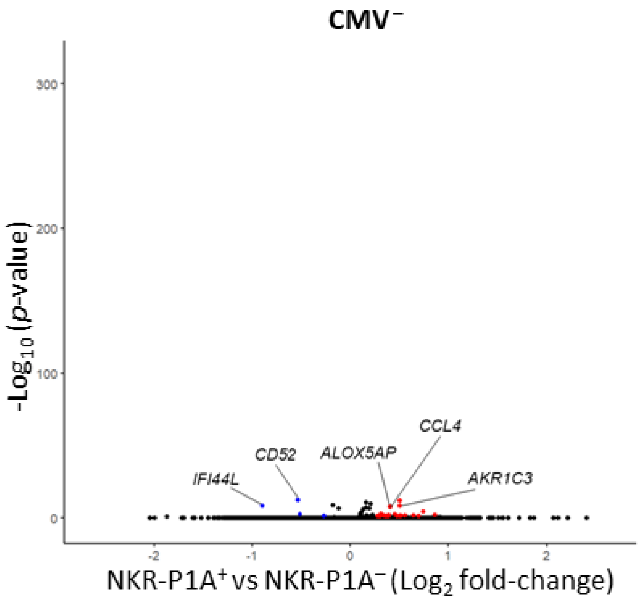

Supplemental Figure 2. Analysis of publicly available NK cell single-cell RNA sequence (scRNA-seq) data. Differentially expressed genes between NKR-P1A$^+$ and NKR-P1A$^-$ NK cells from CMV$^-$ donors.
